# The Psychometric Properties of the Grit-O Scale Within the Twente Region in Netherlands: An ICM-CFA vs. ESEM Approach

**DOI:** 10.3389/fpsyg.2020.00796

**Published:** 2020-05-07

**Authors:** Llewellyn E. van Zyl, Chantal Olckers, Lara C. Roll

**Affiliations:** ^1^Department of Industrial Engineering, University of Eindhoven, Eindhoven, Netherlands; ^2^Optentia Research Focus Area, North-West University (VTC), Vanderbijlpark, South Africa; ^3^Department of Human Resource Management, University of Twente, Enschede, Netherlands; ^4^Institut für Psychologie, Goethe University, Frankfurt am Main, Germany; ^5^Department of Human Resource Management, University of Pretoria, Pretoria, South Africa; ^6^Department of Applied Psychology, Lingnan University, Tuen Mun, Hong Kong

**Keywords:** confirmatory factor analysis, exploratory structural equation modeling, grit, invariance testing, psychometric properties, validity

## Abstract

The purpose of this study was to examine the psychometric properties (i.e., factorial validity, measurement invariance, and reliability) of the Grit-Original scale (Grit-O) within the Netherlands. The Grit-O scale was subjected to a competing measurement modeling strategy that sequentially compared both independent cluster model confirmatory factor analytical- and exploratory structural equation modeling approaches. The results showed that both a two first order, bi-factor structure as well as a less restrictive two factor ESEM factorial structure best-fitted the data. The instrument showed to be reliable at both a lower- (Cronbach’s alpha) and upper-level (composite reliability) limit. However, measurement invariance between genders could only be established for the B-ICM-CFA model. Finally, concurrent validity was established through relating the GRIT-O to task performance. The linear use of the Grit-O scale should therefore carefully be considered.

## Introduction

*Grit*, a universal predictor of life success regardless of context, individual character or occupation, is defined as the trait-level perseverance and passion an individual has to pursue long-term goals (Duckworth et al., 2007). Although the literature describes several other predictors of life success or “achievement” (e.g., intelligence, academic performance, attitudes and aspirations, and personality traits) (Credé et al., 2017), grit signifies strength of character as it encompasses great effort, deep commitment and interest in achieving goals over long periods of time despite setbacks, failure and adversity (Duckworth and Quinn, 2009). Gritty people rarely get tired or distracted from their goals and they can easily adapt to setbacks (Ceschi et al., 2016), whereas others may already have proverbially “given up” in similar scenarios (Akin and Arslan, 2014).

Further, grit is associated with important positive individual and organizational outcomes that involve persistence in pursuing set goals, such as higher efficacy and retention (Duckworth and Quinn, 2009; Lee and Duckworth, 2019), greater work engagement and fewer career changes (Eskreis-Winkler et al., 2014), lifetime educational attainment (Duckworth et al., 2007) and less counter-productive work behaviors (Ceschi et al., 2016). Ceschi et al. (2016) in their study found that grit is accountable for making individuals less vulnerable to the effects of stressful events and impacts performance. Duckworth et al. (2007) and Duckworth and Quinn (2009) showed that grit predicts teacher effectiveness and achievement in academic and avocational domains. Given that grit is an important factor contributing to personal achievement and that it predicts success more efficiently than mere talent (Duckworth et al., 2007; Duckworth and Quinn, 2009), it is not surprising that the concept has gained significant traction in the mass media and popular press.

After Duckworth (2013) TED talk in 2013 on “Grit,” the concept was popularized in the United States as the new “gold standard” for predicting personal and job-related success (Berkowitz, 2016). In her TED talk, Duckworth (2013) argued that grit is more important than talent or skills when it comes to achieving long-term goals. She further argued that if individuals are able to develop “grit,” they will be able to outperform, become more successful and achieve more than their intellectually gifted counterparts (Duckworth, 2016). Although an exaggeration of her academic research findings on grit, this argument seems to have sparked mass-media interest and resulted in the publication of Duckworth (2016) best-selling book ‘*Grit: The Power of Passion and Perseverance*’ (Berkowitz, 2016).^1^ In this manuscript Duckworth (2016) provided a detailed account of her research and provided individuals with various self-development tools and strategies to enhance grit. These strategies are based on an individual’s self-reported level of grit that was argued to be validly and reliably measured by the accompanying “Grit-O” scale (Credé et al., 2017). Both her TED talk and book have led to several other internationally best-selling popular psychology or self-development books that employed the Grit-O scale to aid individuals diagnose and develop grit (cf. Miller, 2017; Sinclair, 2017; Fiore, 2018; Willis, 2018), especially within the Netherlands (Waals, 2016; New York Times, 2019).

Despite its world-wide popularity as a self-assessment tool it’s surprising that only a limited number of academic publications besides Duckworth et al. (2007) original study, examined the validity and reliability of the Grit-O scale. These studies have reported different results regarding the factorial structure, the internal consistencies, and predictive capacity of the instrument (Ceschi et al., 2016; Ion et al., 2017). Given Grit-O’s popularity in the popular press and the limited scientific studies on the scale’s validity outside of the context in which it was developed, further investigation of the psychometric properties of the scale outside the US is needed. Finally, the ability of the Grit-O to predict performance specifically in the work context needs to be investigated.

### The Conceptualization and Measurement of Grit

Duckworth et al. (2007) conceptualized grit as a non-cognitive trait that aids one to feel enduringly passionate and persistent in achieving long term goals. Grit does not estimate the propensity to be a “hard-worker,” but rather refers to the level of determination one exerts in achieving long term goals despite the inherent and associated setbacks and difficulties one might face (Holdan et al., 2018). From this perspective, grit is comprised of two separate, yet related dimensions: (a) perseverance of effort and (b) consistency of interest (Duckworth and Quinn, 2009). Perseverance of effort refers to an individual’s innate ability to exert high levels of sustained or enduring long-term effort to pursue a personal or professional goal despite being confronted with setbacks or failures (Duckworth et al., 2007). Consistency of interest, on the other hand, refers to an individual’s tendency to maintain focused interest in a personal or professional goal over time (Duckworth et al., 2007).

Showing high levels of perseverance and being consistently interested in a given goal over extended periods of time are essential components for success and achievement; which transcends individual talent or intelligence (Duckworth, 2016). Gritty individuals perform better academically (Duckworth et al., 2007), are more effective in their work-related tasks (Robertson-Kraft and Duckworth, 2014), perceive to have more meaning in their lives (Kleiman et al., 2013), are more committed to organizations (Eskreis-Winkler et al., 2014), perform better (Jachimowicz et al., 2018), and report higher levels of wellbeing (Disabato et al., 2018).

In order to measure trait-level grit, its components and how it relates to these aforementioned positive outcomes, Duckworth et al. (2007) developed the 12 item Grit-O scale. The scale measured both perseverance of effort^2^ (six items) and consistency in interest^3^ (six items) in a short, self-report manner. This questionnaire was later shortened to eight items, which Duckworth and Quinn (2009) called the Short Grit scale (Grit-S). The eight-item Grit-S has become a popular tool to measure grit across nations. The Grit-S scale has been the subject of a few validation studies and has been translated into, for example, German (Schmidt et al., 2017), Spanish (Arco-Tirado et al., 2018), and Polish (Wyszyńska et al., 2017). Within applied studies, the Grit-S scale has successively produced various factor structures ranging from an overall one factor model, to a three-factor model (Datu et al., 2015; Hatchimonji, 2016). These studies have also some found significant variability in the reliability of the instrument, which range from poor to acceptable. Weston (2014) criticized the Grit-S scale for low parsimony due to the limited number of items on each subscale, and that it may reflect more error variance than construct variance. She argued that the 12 item Grit-O scale might be more appropriate for future use.

In contrast to the Grit-S, the Grit-O showed more promise as an instrument. In the original Duckworth studies, it consistently showed to be a valid and reliable tool to measure overall grit (Duckworth et al., 2007). However, in the handful of studies in which it was used where Duckworth was not a co-author, the instrument showed different factor structures and reliabilities. Further, in most of these samples the Grit-O was used only within a mono-cultural context and therefore it might not be sensitive to cultural nuances (Credé et al., 2017). According to Disabato et al. (2018), grit, when seen as a psychological strength, is embedded in the values and beliefs of a given culture and is therefore culture-bound. Therefore, both the construct and its measurement may look different in different cultural contexts (Templin and Henson, 2010).

We have identified several gaps in the research that we would like to investigate in our study. First, we could not establish the existence of studies that determined the psychometric properties of the Grit-O when used in contexts other than the United States (Christensen and Knezek, 2014), Russia (Tyumeneva et al., 2017), and South Korea (Kim and Lee, 2015). Specifically, we established that no study has been published that investigated psychometric properties of the Grit-O scale within the Netherlands or in any other Western-European context. Secondly, various research studies have reported different factorial models and differences in internal consistency of the Grit-O and the dimensionality of the Grit-O thus requires additional verification (Tyumeneva et al., 2017). These limited available literature on the psychometric properties of the Grit-O scale further only focused on the traditional confirmatory factor analytical models and failed to investigate the bi-factor structure of Grit and/or any exploratory structural equation models. Therefore, evidence of the Grit-O’s factorial validity, measurement invariance, internal consistency and concurrent validity seems to be severely lacking and needs further investigation.

### Factorial Validity

It is, however, important to establish the factorial validity of an instrument such as the Grit-O to establish whether this instrument truly measures the attribute, grit (Sartori and Pasini, 2007). Research showed that the Grit-O scale produced various item and factor loadings as well as factor structures within various samples. During the original development of the Grit-O, Duckworth et al. (2007) used a US sample, and through a traditional independent cluster modeling confirmatory factor analysis (ICM-CFA), they confirmed the Grit-O as consisting of two factors (i.e., perseverance of effort and interest) in Study 1. However, Duckworth et al. (2007) could only produce a single first order factor structure (overall grit) in the subsequent studies in the same paper. Similarly, Christensen and Knezek (2014) in the United States, and Tyumeneva et al. (2017) in Russia, were able to confirm the original two-factor structure of the Grit-O. However, even when the two-factor structure was confirmed within the Russian study, all the items did not load onto their *a priori* theoretical factors. Tyumeneva et al. (2017) found that item three of the original perseverance-of-effort subscale (“Setbacks do not discourage me”) loaded statistically significantly on the consistency-of-interest subscale of the instrument. In this instance, the perseverance subscale consisted of five items and the consistency-of-interest subscale of seven. Furthermore, their results demonstrated that the Grit-O scale measures two different constructs, consistency-of-interests and perseverance-of-effort rather than the common trait “grit.” In psychometric terms, both constructs therefore differ in respect to what and how they are measured within the Russian context, compared to the United States and South Korea. Meaningful comparisons with the two-factor structure can therefore not be made between these cultural contexts (Templin and Henson, 2010).

In contrast to the two-factor structure, Kim and Lee (2015) found evidence for a three-factor model. Within the South Korean context, the persistence-of-effort subscale produced two latent factors: persistence of effort and industriousness. They argued that the industriousness subscale, which comprised two items (“I am a hard worker” and “I am diligent”), was a separate factor in the South Korean context. According to them, South Koreans perceived industriousness (i.e., the innate ability to work very hard and diligently) as a separate factor contributing to grit.

Similarly, Disabato et al. (2018) research on an international sample from six continents affirmed a bifactor model of the Grit-O, conceptually similar to a hierarchical CFA, of the Grit-O. Their study confirmed the multidimensionality of the Grit-O, suggesting that the items measured reflect multiple constructs of grit as a broad trait (overall grit) and as a specific facet (perseverance and interest). Despite this single study employing a less restrictive CFA model, no other studies could be found that employed even less restrictive models, such an Exploratory Structural Equation Modeling (ESEM) approaches, to investigate the factorial validity of the Grit-O.

Therefore, it is not clear whether a more restrictive (ICM-CFA) or less restrictive (ESEM) model would be better suited for estimating grit via the Grit-O scale. Further, it is also not clear if the Grit-O scale will show factorial validity within other Western contexts, despite its wide-spread use within practice. The question we therefore asked is how the factor structure manifest in a Western-European context such as the Netherlands. Similarly, would a more restrictive or less restrictive factorial model be preferred?

### Internal Consistency

Although previous studies found the Grit-O to be a reliable measurement instrument (Duckworth et al., 2007; Kim and Lee, 2015), the level of internal consistency varied significantly across samples. Studies using a two-factor model for the Grit-O reported acceptable Cronbach’s alpha values for the overall scale (α = 0.85) and for each dimension (perseverance of effort, α = 0.78; interest, α = 0.84) (Duckworth et al., 2007). The study of Christensen and Knezek (2014) revealed the following Cronbach’s alpha values: overall scale, α = 0.85; perseverance of effort, α = 0.68; interest, α = 0.74. Kim and Lee (2015) study, which assumed the Grit-O as a three-factor structure, reported Cronbach’s alpha values of 0.79 (interest), 0.76 (persistence of effort), and 0.84 (industriousness). In these studies, Cronbach’s alphas were calculated as reliability indicators. The use of Cronbach’s alpha often results in over- or underestimating reliability, being based on the assumption that the factor loadings and error variances are equal (Doré et al., 2017).

Studies that considered the Grit-O as a bifactor model calculated omega reliability coefficients in the final permutation. The explained common variance of the overall grit factor was 0.49, lower than the 0.60 cut-off (Rodriguez et al., 2016), whereas interest explained common variance of 0.74, and perseverance explained common method variance of 0.60. The current study aimed to estimate both the Cronbach’s alpha as well as composite reliability rho, measuring levels of variance caused by a measurement instrument in relation to variance caused by random measurement error and correcting for over- or underestimating reliability. This study hypothesized that Grit-O presented acceptable levels of internal consistency at both the lower- (Cronbach’s alpha ≥ 0.70) and upper- (composite reliability/rho coefficients > 0.70) level limits.

### Measurement Invariance

Research has shown that grit is an intra-personal psychological strength that varies between individuals, across generational cohorts and between genders (Christensen and Knezek, 2014; Clark and Malecki, 2019). Studies showed that high-school students report higher levels of grit than middle-school students (Cosgrove et al., 2016), older working adults tended to be grittier than younger ones (Duckworth et al., 2007) and young female adults had slightly higher levels of grit than their male counterparts (Christensen and Knezek, 2014). When evaluating the two components of grit separately, one study showed that females tended to have higher levels of interest than males, whereas males had higher levels of perseverance (Christensen and Knezek, 2014). These group-level gender-related differences in grit could potentially influence how males and females perceive grit, which could subsequently influence how it is measured.

Another factor to consider when measuring grit is gender bias in psychological assessment (Reynolds and Suzuki, 2012; Willingham and Cole, 2013). Various studies showed that self-report psychological assessments (particularly personality and cognitive assessments) inherently discriminate between genders (Lindsay et al., 2000; Willingham and Cole, 2013; Brabender and Mihura, 2016; Krishnamurthy, 2016). Psychometric tools developed within WEIRD (White Educated, Industrialized, Rich, and Democratic) contexts tend to inherently favor males over females (Ludeke and Larsen, 2017; Fernandez, 2019). Newly developed or poorly used self-report assessment measures (such as the Grit-O scale) are more prone to gender-related bias, as they have been subjected to less scientific scrutiny.

Given the significant differences between genders regarding grit and increased use of the Grit-O scale in academic literature (and within mass media), it is imperative to investigate the measurement invariance thereof to ensure that gender-related measurement bias is ruled out. Although Duckworth and Quinn (2009) established measurement invariance across genders using Grit-S in six different studies, only one study established measurement invariance on the Grit-O (see Christensen and Knezek, 2014). This study showed evidence of measurement invariance among young adults of different genders. As such, we hypothesized that the Grit-O scale will show configural, metric and scalar measurement invariances between genders.

### Concurrent Validity

The main function of girt is that it’s a precursor for performance (Jachimowicz et al., 2018). Grit is positioned as a vital personal resource required to translate individual drive and resolve, into measurable performance outcomes on both an individual and organizational level (Nelson and Baltes, 2019). Previous studies reported a direct and positive relationship between grit and various permutations of performance ranging from academic success/performance (Duckworth and Quinn, 2009; Jachimowicz et al., 2018; Nelson and Baltes, 2019), and training performance in sports (Cazayoux and DeBeliso, 2019), to job performance (Jordan et al., 2019; Kim et al., 2019; Webster-Wright, 2019), operational productivity (Steuber et al., 2019), and even task performance (Vogelsang, 2018). Koopmans et al. (2013) argued that task performance seems to be an important indicator of operational efficiency and personal performance, which is also strongly influenced by non-cognitive traits such as personality, interest and drive. From this perspective, task performance is defined as the proficiency with which individuals perform the most important or core substantive tasks that is central to their jobs (Koopmans et al., 2013).

It has been argued that gritty individuals are better equipped to utilize their capabilities in order to perform their work-related tasks which are aligned to their interests (Vogelsang, 2018). Gritty individuals therefore prioritize the completion of short-term tasks through broadly relating such to their personal and professional long-term goals (Vogelsang, 2018). These individuals are therefore also less likely to be affected setbacks and therefore more focused on performing their work-related tasks well (Steuber et al., 2019). Given strong association between Grit and Task Performance, it could provide an adequate means through which to establish concurrent validity of the Grit-O scale.

### The Present Study

Given the inconsistency in the factor structures and reliabilities produced by the Grit-O scale, the structure of such cannot accurately be estimated by the traditional independent cluster model confirmatory factor analytical (ICM-CFA) approaches (Morin et al., 2013). This highly restrictive ICM-CFA approach forces items to only load onto their *a prior* theoretical factor, where factor loadings on other constructs are constrained to zero (Marsh et al., 2011). When many of the factor loadings are constrained to zero, it results in poor model fit and an over-estimation or inflation of factor correlations (Marsh et al., 2011). This in turn not only results in fewer distinct factors but leads to potential measurement bias (Wang and Wang, 2012; Gucciardi and Zyphur, 2016). We started our analyses by testing several of these restrictive models (ICM-CFA) of the Grit-O. First, we tested whether the data fit a single-factor (overall) grit model, as were found by Duckworth et al. (2007) in her study. Second, based on the studies conducted by Christensen and Knezek (2014) and Tyumeneva et al. (2017), we tested whether the Grit-O scale is multidimensional as it comprises two dimensions (perseverance and interest). Based on the research findings by Kim and Lee (2015), we also tested for a three-factor structure of the grit scale. A hierarchical second-order factor model was tested to explore whether the two first-order factors (perseverance and interest) would load onto a higher-order grit factor as were originally theorized by Duckworth et al. (2007). Even after correlated the error terms of several items when testing a one as well as two-factor structure of the Grit-O scale, Disabato et al. (2018), model fit remained unacceptable and they therefore tested and confirmed a bifactor model. Based on their findings, we also tested a bifactor model to establish whether the scale items reflect multiple constructs, both an overall, broad grit factor and two specific dimensions/facets. Testing a bifactor model allows for significant tests of the overall factor or specific factors above and beyond the other(s) (Chen et al., 2012).

Because of this over-inflation of factor correlations, Morin et al. (2013) recommended the use of ESEM to estimate the factor structure of multi-dimensional constructs such as grit. ESEM incorporates an exploratory function within the traditional ICM-CFA framework, where all cross-loadings are freely estimated but could be targeted and constrained (Asparouhov and Muthén, 2009). This results in better fitting models that are able to provide more distinction between factors. Therefore, less restrictive ESEM models may be superior to ICM-CFA models when attempting to capture the structural dimensionality of multi-factor instruments (Joshanloo and Weijers, 2019) such as grit. To obtain acceptable fit for their bifactor model, Disabato et al. (2018) correlated the error terms of several items, therefore we have decided to test for a less restrictive ESEM model of the Grit-O. Testing an ESEM model will provide us more flexibility in evaluating the factor structure of the grit-scale as it allows for cross-loadings in the model. As originally theorized by Duckworth et al. (2007), the Grit-O include both sources of construct-relevant multidimensionality, as this scale assessed the presence of both overall grit and two specific facets, perseverance and effort.

Given that different factorial models and differences in internal consistency were reported across different studies, an ESEM model may yield both better model-fit and produce more accurate inter-factor correlations versus its ICM-CFA counterpart. However, testing ICM-CFA models are still required in order to make meaningful comparisons to previous research. Therefore, both ESEM (first order ESEM, and Bifactor ESEM) and ICM-CFA (first- and second order- and bifactor) models need to be assessed when determining the factorial validity of the Grit-O.

The purpose of our study is to examine the psychometric properties of the Grit-O scale by determining its factorial validity, reliability, measurement invariance, and concurrent validity. The contribution of this study is twofold: (1) Firstly, to contribute to the body of knowledge regarding the dimensionality and reliability of the Grit-O specifically when applied in a Western-European context by comparing various traditional confirmatory factor analytical models with less restrictive ESEM models and (2) to provide empirical evidence on the ability of the Grit-O to predict task performance within the work context.

## Materials and Methods

### Research Design

The study employed a descriptive, quantitative, cross-sectional survey-based research design to determine the psychometric properties of the Grit-O scale when used on a sample from the Twente region in the Netherlands. This design provided a means to measure grit at a single point in time (i.e., by using timestamping) in order to determine the psychometric properties of the Grit-O scale.

### Research Procedure

This study formed part of a larger research project on grit within the Twente region in the Netherlands. The researchers obtained permission from the research institution’s research ethics committee to conduct the study. We recruited five small- and medium-sized enterprises (SMEs) in the Twente region to participate in the study, and collected data through a self-administered online questionnaire. The study also measured demographic information, biographic characteristics and self-reported English language proficiency. Each questionnaire included a cover letter inviting individuals to participate voluntarily and anonymously. It provided a detailed explanation of the research procedure, the potential risks, discomforts and benefits associated with participation, and highlighted the rights and responsibilities of all parties involved. Respondents were assured that their responses would remain confidential and would be used for research purposes only. We discussed with respondents the voluntary nature of the study and their right to withdraw. Respondents received no payment or incentive to encourage participation. They had to agree to the terms of participation in order to complete the questionnaire. We sent an invitation email with the link to the online survey to the contact persons at the various SMEs for distribution to their internal networks. During the 3-week data collection process, we sent two reminder emails encouraging participation.

We stored the data on a secure SQL server, and scrubbed the meta-data before downloading and processing the data set. We screened the data of the sample of 401 respondents who had completed the survey and excluded from the analysis those who reported below average levels of English proficiency and those who had given incomplete responses. In total, 90 respondents were excluded.

### Participants

Using a convenience sampling strategy, we drew 311 employed respondents from the Twente region in the Netherlands to participate in this research. The region is bordered by Germany on the east, and its working population is comprised mainly of Dutch and German nationals. However, it also draws many highly skilled migrants from across Europe and other continents (Statistics Netherlands, 2016). Respondents’ ethnicity, age, years of employment, and educational information are summarized in Table 1.

**TABLE 1:** Demographic and biographic characteristics.

Variable	Category	Frequency (*f*)	Percentage (%)
Gender	Male	92	29.6
	Female	215	69.1
	Missing or prefer not to be identified	4	1.3
Age in years	18–20	39	12.5
	21–30	134	43.1
	31–40	62	19.9
	41–50	21	6.8
	51–60	30	9.6
	61+	12	3.9
	Missing or prefer not to be identified	13	4.2
Native language	English	43	13.8
	Dutch	23	7.4
	German	195	62.7
	Other	50	16.1
Nationality	Dutch	24	7.7
	German	199	64.0
	South African	59	19.0
	Other (European)	29	9.3
Level of education	Did not complete high school	9	2.9
	High school	90	28.9
	Diploma	23	7.4
	Bachelor’s degree	68	21.9
	Master’s degree	68	21.9
	Advanced graduate work or Ph.D.	49	15.8
	Missing or prefer not to be identified	4	1.3
Years of employment in current position	0–5	239	76.8
	6–10	32	10.3
	11–15	16	5.2
	16+	15	4.8
	Missing or prefer not to be identified	9	2.9

The majority of the participants were German-speaking (62.7%) females (69.1%) of German descent (64.0%) between the ages of 21 and 30 years (43.1%). Most of the sample had completed at least a high school level of education (28.9%) and had worked between 0 and 5 years (76.8%) in their current position.

### Measures

This study used the following three instruments to gather data:

A biographical questionnaire was used to gather biographic information about the participants and assess their level of English proficiency.

The Grit-O scale developed by Duckworth et al. (2007) was used to measure grit. The 12-item questionnaire measured the two components interest (six items, e.g., “My interests change from year to year”) and perseverance (six items, e.g., “I have overcome setbacks to conquer an important challenge”) – on a 5-point Likert scale ranging from 1 (“Not like me at all”) to 5 (“Very much like me”). All the items on the consistency-of-interest subscale were reverse-coded (items 2, 3, 5, 7, 8, and 11). The Grit-O scale showed acceptable levels of internal consistency with Cronbach’s alphas of 0.84 on both scales (Duckworth et al., 2007).

The *Task Performance Subscale* of the Individual Work Performance Scale developed by Koopmans et al. (2013) was employed to measure task performance by means of seven items on a 6-point Likert scale ranging from 1 (“Never”) to 6 (“Always”). An example of an item is: “I kept in mind the results that I had to achieve in my work.” Van Zyl et al. (2019) found acceptable levels of internal consistency for the instrument with a Cronbach’s alpha level of 0.88.

### Statistical Analyses

Data was processed with Mplus version 8.3 (Muthén and Muthén, 2017). First, we estimated factorial validity through a competing measurement modeling strategy with the maximum likelihood estimator. Both traditional independent cluster model confirmatory factor analytical- (ICM-CFA: first, second and bifactor) and ESEM (first order ESEM, and Bifactor ESEM) models were estimated and sequentially compared. For the ICM-CFA models, items were only permitted to load onto their *a priori* theoretical factor and cross-loadings were constrained to zero. For the BiFactor models (B-ICM-CFA) an orthogonal targeted rotation was employed. Here, a general factor (G-Factor) of overall grit was specified which was comprised of all the items of the Grit-O scale. Further, two specific factors (S-Factors), corresponding to the *a priori* interest and perseverance theoretical dimensions, were specified. For the ESEM models, a targeted rotation was again used. Cross-loadings of items were permitted but constrained to be close to zero (Brown, 2006). Again, items were specified to load on their *a priori* theoretical constructs. For the Bifactor ESEM model (B-ESEM), a similar strategy to the B-ICM-CFA models was employed. However, cross-loadings were permitted and targeted to be as close to zero as possible. For all the models, observed items were used as indicators for latent variables. De Beer and Van Zyl (2019) ESEM code generator for Mplus was used to generate the syntaxes for these models. To estimate model fit and to compare competing measurement models, the commonly used fit statistics and information criteria for structural equation modeling suggested by Wang and Wang (2012) were used. Table 2 indicates the fit indices and cut-off values used to determine model fit.

**TABLE 2:** Fit indices: acceptable values and cut-off points.

Fit indices	Acceptable values
**Absolute fit indices**	
Chi-square	Lowest value in comparative measurement models
Root Mean Square Error of Approximation (RMSEA)	0.06–0.08 (Marginally Acceptable); 0.01–0.05 (Excellent)
Standardized Root Mean Square Residual (SRMR)	0.06–0.08 (Marginally Acceptable); 0.01–0.05 (Excellent)
**Incremental fit indices**	
Comparative Fit Index (CFI)	0.90–0.95 (Marginal Fit); 0.96–0.99 (Excellent Fit)
Tucker–Lewis Index (TLI)	0.90–0.95 (Marginal Fit); 0.96–0.99 (Excellent Fit)
Akaike Information Criterion (AIC)	Lowest value in comparative measurement models
Bayes Information Criterion (BIC)	Lowest value in comparative measurement models
Sample-Size Adjusted BIC (aBIC)	Lowest value in comparative measurement models

*Source: Wang and Wang (2012).*

Second, once the best fitting measurement models were identified, the standardized item loadings (λ > 0.30; *p* < 0.01), standard errors and item uniqueness were inspected to further discriminate between models (Asparouhov and Muthén, 2009).

Third, both internal consistencies and the intercorrelations between factors of the best fitting measurement models were computed. To assess the internal consistency of the Grit-O, the point-estimate composite reliability (ρ_*esem*_ > 0.70) measure of Raykov and Shrout (2002) was used for ESEM factors, as well as rho (ρ > 0.70) (Wang and Wang, 2012) and Cronbach’s alpha (α > 0.70) (Nunnally and Bernstein, 1994) for ICM-CFA factors. Intercorrelations between factors on both ESEM and ICM-CFA models were computed to determine the level of unique distinction between factors. Statistical significance was set at the 95% confidence interval.

Third, we investigated measurement invariance based on gender (males and females), and we computed configural (similar factor structures), metric (similar factor loadings), scalar (similar intercepts), and full uniqueness (constraining all factor loadings, intercepts and residual variances to be equal) invariances. Invariance was determined through a non-significant difference in chi-square between genders (*p* > 0.05), as well as changes in RMSEA (Δ < 0.01), SRMR (Δ < 0.02 for configural vs. metric; Δ < 0.01 metric vs. scalar), and CFI (Δ < 0.01) were indicative of invariance (Chen, 2007; Wang and Wang, 2012).

If we established invariance, we computed and categorically compared the latent mean differences between genders. We identified one group as a reference group (setting its mean at zero), and freely estimated the comparative group’s mean. Should the comparative group’s latent mean differ significantly from zero, the groups are found to differ significantly from one another (Wang and Wang, 2012).

Finally, we estimated *concurrent validity* through converting the best fitting measurement models into structural models, with regressive paths pointing toward task performance. Table 2 will once again be used to estimate model fit. The significance level was set at *p* < 0.05.

## Results

To investigate the psychometric properties of the Grit-O scale and to determine the best fitting measurement model, we reviewed the results relating to factorial validity, internal consistency (reliability), measurement invariance across genders and concurrent validly relating to task performance. The results are presented in a tabulated format followed by a brief interpretation.

### Factorial Validity

We determined the factorial validity of the Grit-O scale through comparing five ICM-CFA models, two Bifactor ICM-CFA models (specified as orthogonal) and two ESEM Factorial Solutions. No items were omitted, and observed/measured items were used as indicators of the latent variables within these measurement models (Wang and Wang, 2012). The following models were systematically and structurally compared and results are presented in Table 3:

**TABLE 3:** Goodness-of-fit statistics and information criteria for the competing measurement models.

Model	Type	χ ^2^	*df*	CFI	TLI	RMSEA	SRMR	AIC	BIC	aBIC
Model 1	ICM-CFA: One Factor	402.85	54	0.62	0.54	0.14	[0.131, 0.157]	0.12	10063.94	10198.57	10084.39
Model 2	ICM-CFA: Two Factor	143.44	53	0.90	0.89	0.07	[0.060, 0.089]	0.07	9806.52	9944.89	9827.54
Model 3	ICM-CFA: Two Factor Second Order	143.44	54	0.90	0.89	0.06	[0.060, 0.089]	0.07	9806.52	9944.89	9827.54
Model 4	ICM-CFA: Three Factor	128.32	51	0.91	0.89	0.07	[0.055, 0.085]	0.07	9795.40	9941.25	9817.55
Model 5	ICM-CFA: Three Factor Second Order	140.13	52	0.91	0.88	0.07	[0.059, 0.089]	0.07	9805.21	9947.32	9826.81
Model 6	B-ICM-CFA 1:	77.28	42	0.96	0.94	0.05	[0.033, 0.070]	0.05	9762.35	9941.86	9789.62
	General Factor										
	Two Factors										
Model 7	B-ICM-CFA 2:	43.54	39	0.99	0.98	0.02	[0.001, 0.045]	0.03	9734.62	9925.35	9763.60
	General Factor										
	Three Factors										
Model 8	ESEM:	74.61	43	0.98	0.97	0.05	[0.029, 0.067]	0.03	9757.68	9933.45	9784.39
	Two Factor										
Model 9	ESEM:	35.96	33	0.99	0.98	0.02	[0.001, 0.046]	0.02	9739.04	9952.21	9771.42
	Three Factor										
Model 10	B-ESEM	35.96	33	0.99	0.98	0.02	[0.001, 0.046]	0.02	9739.04	9952.21	9771.42

*χ^2^, chi-square statistic; df, degrees of freedom; TLI, Tucker–Lewis Index; CFI, Comparative Fit Index; RMSEA, Root Mean Square Error of Approximation; SRMR, Standardized Root Mean Square Residual; AIC, Akaike Information Criterion; BIC, Bayes Information Criterion; aBIC, Sample-size adjusted BIC; p < 0.05.*

(1) **Model 1**: A first order ICM-CFA factorial solution was computed where all 12 items directly loaded onto a first order latent variable called grit.
(2) **Model 2**: A two factor, first order ICM-CFA solution was computed that consisted of a factor called interest (items 2, 3, 5, 7, 8, and 11) and perseverance (items 1, 4, 6. 9, 10, and 12).
(3) **Model 3**: A two factor, second order ICM-CFA model was computed that consisted of two first order factors (specified in Model 2), that loaded onto a second order factor called grit.
(4) **Model 4**: A three factor, first order ICM-CFA factorial model solution was specified consisting of interest (items 2, 3, 5, 7, 8, and 11), perseverance (items 1, 4, 9, and 10), and industriousness (items 6 and 12).
(5) **Model 5**: A three factor, second order ICM-CFA model was computed that consisted of three first order factors (specified in Model 4), that loaded onto a second order factor called grit.
(6) **Model 6:** A Bifactor ICM-CFA (B-ICM-CFA-1) consisting of one general factor of grit (where all 12 items directly loaded onto such) and two specific first order factors (as estimated in Model 2) was specified.
(7) **Model 7:** A Bifactor ICM-CFA (B-ICM-CFA-2) consisting of one general factor of grit (where all 12 items directly loaded onto such) and three specific first order factors (as estimated in Model 3) was specified.
(8) **Model 8**: A less restrictive two factor ESEM model of interest (items 2, 3, 5, 7, 8, and 11) and perseverance (items 1, 4, 6. 9, 10, and 12) was estimated. Cross-loadings were permitted but targeted to be as close to zero as possible.
(9) **Model 9**: A less restrictive three factor ESEM model of interest (items 2, 3, 5, 7, 8, and 11), perseverance (items 1, 4, 9, and 10) and industriousness (items 6 and 12) was estimated. Cross-loadings were permitted but targeted to be as close to zero as possible.
(10) **Model 10**: A Bifactor ESEM (B-ESEM) model with one general factor of grit and two specific factors (as specified in Model 8) were estimated. All 12 of the items were directly loaded onto the general factor. The items on the specific factors were permitted to cross-load, but non-intended cross-loadings were targeted to be as close as zero as possible.

Table 3 indicates that the less restrictive ESEM (Models 8, 9, and 10) and B-ICM-CFA (Models 6 and 7) models provided excellent data fit. However, none of the traditional ICM-CFA models (Models 1, 2, 3, 4, and 5) met all of the model fit criteria specified in Table 2. The BSEM (Model 10) and three factor ESEM model (Model 9) produced similar fit statistics. Both fitted the data comparatively better than the two factor ESEM (Model 8: Δχ^2^ = −38.92; *df* = −10; *p* < 0.01; ΔCFI = −0.01; ΔTLI = −0.01; ΔRMSEA = −0.03; ΔSRMR = −0.01; ΔAIC = −18.64; ΔaBIC = −12,97), and the two specific-factor Bifactor model (B-ICM-CFA 1 Model 6: Δχ^2^ = −41.32; *df* = −9; *p* < 0.01; ΔCFI = −0.03; ΔTLI = −0.04; ΔRMSEA = −0.03; ΔSRMR = −0.01; ΔAIC = −23.31; ΔaBIC = −18.20). Models 9 and 10 did not statistically significantly differ from the three specific-factor Bifactor Model (Model 7 Δχ^2^ = −7.58; *df* = −6; *p* > 0.01; ΔCFI = 0.00; ΔTLI = 0.00; ΔRMSEA = 0.00; ΔSRMR = −0.01; ΔAIC = 4.42; ΔaBIC = 7.82).

Further, the inter-factorial correlations in Models 2 and 3 (interest vs. perseverance: *r* = 0.37, *p* < 0.01), as well as Models 4 and 5 (interest vs. perseverance: *r* = 0.45, *p* < 0.01; industriousness vs. interest: *r* = 0.22, *p* < 0.01 and industriousness vs. perseverance: *r* = 0.87, *p* < 0.01) were less than acceptable (*r* > 0.50 but < 0.90) (Wang and Wang, 2012). This implies that the facets aren’t strongly nor uniformly correlated with one another. Models 1 through 5 are therefore disregarded from further analyses.

Next, the standardized factor loadings, standard errors and item uniqueness for the B-ICM-CFA (Models 5 and 6) and ESEM models (8, 9, and 10) were estimated in order to further establish the factorial validity of the Grit-O. The results are summarized in Table 4.

**TABLE 4:** Standardized factor loadings (λ) and item uniqueness (δ) for the B-ICM-CFA, ESEM and B-ESEM solutions.

Factor	Item	B-ICM-CFA 1 Model 6					B-ICM-CFA 2 Model 7					ESEM Model 8					ESEM Model 9							B-ESEM Model 10						
		G_*factor*_		S_*factor*_			G_*factor*_		S_*factor*_			F1		F2			F1		F2		F3			G_*factor*_		S_*factor 1*_		S_*factor 2*_		
		λ	S.E.	λ	*S.E.*	δ	λ	*S.E.*	λ	*S.E*.	δ	λ	*S.E*.	λ	*S.E.*	δ	λ	*S.E*.	λ	*S.E.*	λ	*S.E.*	δ	λ	*S.E.*	λ	*S.E*.	λ	*S.E.*	δ
**Interest (F1)**	GRIT2	**0.18**	0.06	**0.50**	0.07	0.73	**0.28**	0.13	**0.46**	0.08	0.71	**0.53**	0.05	−0.08	0.05	0.74	**0.53**	0.05	−0.15	0.07	0.17	0.07	0.67	−0.15	0.12	**0.51**	0.06	0.11	0.15	0.67
	GRIT3	**0.20**	0.10	**0.66**	0.05	0.53	0.27	0.17	**0.60**	0.08	0.57	**0.67**	0.04	−0.07	0.06	0.58	**0.65**	0.04	−0.04	0.06	0.07	0.06	0.57	−0.02	0.12	**0.65**	0.04	0.03	0.10	0.57
	GRIT5	**0.44**	0.11	**0.53**	0.09	0.53	0.11	0.16	**0.69**	0.04	0.52	**0.67**	0.04	0.12	0.05	0.52	**0.68**	0.04	0.11	0.06	0.07	0.06	0.52	0.13	0.13	**0.66**	0.04	−0.03	0.09	0.52
	GRIT7	**0.53**	0.10	**0.55**	0.09	0.42	0.01	0.18	**0.76**	0.03	0.42	**0.70**	0.04	0.24	0.05	0.44	**0.73**	0.04	0.26	0.07	−0.05	0.06	0.40	**0.30**	0.11	**0.72**	0.06	0.03	0.09	0.40
	GRIT8	**0.59**	0.09	**0.33**	0.12	0.54	−0.17	0.14	**0.65**	0.06	0.56	**0.51**	0.05	0.07	0.05	0.61	**0.56**	0.05	0.35	0.06	−0.02	0.07	0.57	**0.37**	0.11	**0.53**	0.07	0.06	0.10	0.57
	GRIT11	**0.20**	0.02	**0.62**	0.06	0.61	0.35	0.13	**0.50**	0.09	0.63	**0.64**	0.05	−0.22	0.05	0.63	**0.59**	0.05	−0.12	0.06	−0.08	0.07	0.63	−0.08	0.10	**0.62**	0.05	−0.13	0.08	0.63
**Persever**	GRIT1	**0.44**	0.11	**0.39**	0.11	0.65	−**0.63**	0.13	**0.35**	0.17	0.48	−0.10	0.05	**0.65**	0.06	0.61	0.01	0.05	**0.74**	0.06	0.09	0.06	0.45	**0.70**	0.10	−0.09	0.05	0.26	0.06	0.45
**ance (F2)**	GRIT4	**0.32**	0.10	**0.31**	0.10	0.81	−**0.38**	0.10	**0.29**	0.11	0.77	−0.06	0.06	**0.48**	0.05	0.78	0.04	0.06	**0.43**	0.06	0.18	0.07	0.78	**0.38**	0.09	−0.05	0.06	0.28	0.05	0.78
	GRIT9	**0.51**	0.07	**0.39**	0.08	0.59	−0.19	0.16	**0.64**	0.07	0.55	0.22	0.05	**0.69**	0.04	0.58	0.33	0.05	**0.41**	0.05	**0.39**	0.07	0.57	**0.34**	0.13	0.21	0.05	**0.62**	0.05	0.57
	GRIT10	**0.54**	0.09	**0.35**	0.10	0.58	−**0.35**	0.14	**0.54**	0.09	0.59	0.12	0.05	**0.55**	0.05	0.59	0.23	0.05	**0.52**	0.06	**0.27**	0.06	0.60	**0.46**	0.11	0.13	0.05	**0.47**	0.05	0.60
	GRIT6	**0.31**	0.09	**0.65**	0.07	0.48	–	–	–	–	–	−0.09	0.05	**0.59**	0.05	0.55	–	–	–	–	–	–	–	**0.36**	0.12	−0.11	0.04	**0.38**	0.06	0.51
	GRIT12	**0.27**	0.09	**0.60**	0.07	0.57	–	–	–	–	–	−0.01	0.05	**0.59**	0.05	0.65	–	–	–	–	–	–	–	0.21	0.14	−0.05	0.05	**0.67**	0.06	0.52
**Industrious**	GRIT6	–	–	–	–	–	−**0.35**	0.14	**0.61**	0.10	0.51	–	–	–	–	–	0.05	0.05	**0.48**	0.06	**0.51**	0.08	0.51	–	–	–	–	–	–	–
** ness (F3)**	GRIT12	–	–	–	–	–	−0.16	0.14	**0.69**	0.06	0.5	–	–	–	–	–	0.11	0.05	**0.34**	0.06	**0.60**	0.08	0.52	–	–	–	–	–	–	–

*Bold items loaded significantly (p < 0.05); Underlined items indicate cross-loading items; S.E., standard error.*

For both the two factor B-ICM-CFA-1 and the two factor ESEM model (Model 8), the items loaded sufficiently and statistically significantly (λ > 0.30; *p* < 0.01) on each of their *a prior* theoretical factors. For the B-ICM-CFA-1 Model (Model 6), all the items loaded significantly on the General Factor [λ_(General factor)_ = 0.30–0.59; *p* < 0.05; Mean λ = 0.39] as well as on both the Specific Factors [Interest: λ_(Specific factor)_ = 0.33–0.66; *p* < 0.05; Mean λ = 0.53; Perseverance of Effort: λ_(Specific factor)_ = 0.31–0.65; *p* < 0.05; Mean λ = 0.45]. This implies that Model 6 produced a well-defined G-Factor representing overall grit. Similarly, the less restrictive two factor ESEM model (Model 8) showed that items loaded sufficiently and statistically significantly on their *a priori* theoretical Interest (λ = 0.53–0.67; *p* < 0.05; Mean λ = 0.62) and Perseverance (λ = 0.48–0.69; *p* < 0.05; Mean λ = 0.59) factors. Here, no significant cross-loadings were present.

However, although the remaining models (Models 7, 9, and 10) produced sufficient model fit, not all items loaded sufficiently or statistically significantly on their respective theoretical factors. First, the three factor B-ICM-CFA-2 model (Model 7) did not produce a significant General Factor, where only 4 items (GRIT1: λ = −0.63; GRIT2 λ = −0.28, GRIT4 λ = −0.38, GRIT10 λ = −0.35) loaded significantly onto such. Moreover, the item loading for the perseverance item GRIT4 was below the suggested 0.30 cutoff. However, besides GRIT4, all other items for the interest (λ = 0.46–0.76; *p* < 0.05; Mean λ = 0.61), perseverance (λ = 0.35–0.46; *p* < 0.05; Mean λ = 0.51), and industriousness (λ = 0.61–0.69; *p* < 0.05; Mean λ = 0.65) subscales loaded sufficiently and significantly. Therefore Model 7 was disregarded from further analyses.

Further, although all the items for the three factor ESEM model (Model 9) loaded sufficiently and significantly (interest: λ = 0.53–0.73, *p* < 0.05, Mean λ = 0.62; perseverance of effort: λ = 0.41–0.74, *p* < 0.05, Mean λ = 0.49; and industriousness = λ = 0.51–0.61, *p* < 0.05, Mean λ = 0.55) on their *a priori* theoretical constructs, items GRIT6 and GRIT12 produced statistically significant and practically sufficient cross-loadings between the perseverance and industriousness factors. Although cross-loadings are to be expected and allowed within the ESEM framework, larger cross-loadings may provide an indication that a conceptual overlap between items and factors exists (Morin and Maïano, 2011). Therefore, Model 9 was disregarded from further analyses.

Finally, the Bifactor ESEM model (B-ESEM Model 10) did not produce a statistically significant General Factor. Five items (GRIT2, GRIT3, GRIT5, GRIT11, and GRIT12) did not load statistically significantly on the G-Factor (λ > 0.30; *p* < 0.01) and neither did item GRIT1 and GRIT4 on their *a priori* perseverance specific factor. This indicates that a General Factor for grit within a less restrictive framework is not present. Therefore, Model 10 was also disregarded from further analyses. As such, only Models 6 and 8 were retained for further analyses.

### Factor Intercorrelations and Internal Consistencies

Factorial intercorrelations and internal consistencies for the ESEM and B-ICM-CFA-1 factors were computed (cf. Table 5). Internal consistency estimation showed both the ESEM and B-ICM-CFA factors were reliable at both an upper and lower level limit (ρ > 0.80: Raykov, 2009; α > 0.70: Nunnally and Bernstein, 1994; ρ_*esem*_ > 0.70: Raykov and Shrout, 2002). Further, the factor correlations showed that perseverance and consistency of interest on the ESEM model (*r* = 0.28; *p* < 0.01) produced statistically significantly smaller correlations than the B-ICM-CFA-1 model (*r* = 0.56; *p* < 0.01). This implies that the ESEM model is able to provide a slightly better distinction between the components of grit than the B-ICM-CFA-1 model. Therefore, both models are retained for further analyses.

**TABLE 5:** Factor correlations and internal consistencies of the factors for both the B-ICM-CFA (Model 6) and ESEM Solution (Model 8).

No	Variable	ρ	α	ρ_*esem*_	1	2	3
(1)	Interest	0.79	0.79	0.73	–	0.28	0.00
(2)	Perseverance	0.78	0.76	0.71	0.56	–	
(3)	General factor (overall grit)	0.77	0.79	–	0.00	0.00	–

*B-ICM-CFA correlations are shown below the diagonal; ESEM correlations are shown above the diagonal. ρ = composite reliability (rho); α = Cronbach’s alpha. ρ_esem_ all factors related statistically significantly (p < 0.05).*

### Measurement Invariance

Next, measurement invariance across genders (males: 92 vs. females: 211) was computed for both the B-ICM-CFA-1 and the ESEM model. First, the Kaiser–Meyer–Olkin (KMO) measure of sampling adequacy computed to determine whether the sample-size was sufficient to compute invariance for each gender. The results showed that the sample sizes for both genders were adequate (KMO < 0.70, *p* < 0.01) (Cerny and Kaiser, 1977) and therefore measurement invariance can be computed.

Table 6 shows that for the B-ICM-CFA-1 model invariance could be established across genders. Non-significant differences in χ^2^ and changes smaller than 0.01 in CFI between the configural, metric, and scalar invariance models (*p* > 0.01) were found. Further, the differences in RMSEA (Δ < 0.01) and SRMR (Δ < 0.02 for configural vs. metric; Δ < 0.01 metric vs. scalar) were below the specified levels (Chen, 2007; Wang and Wang, 2012). Therefore, measurement invariance for the B-ICM-CFA-1 factor was established.

**TABLE 6:** Invariance testing between the different genders for the B-ICM-CFA Model (Model 6).

	Model	χ ^2^	*df*	CFI	TLI	RMSEA		SRMR	AIC	aBIC	Model Comparison	Δχ ^2^	Δ CFI	Δ RMSEA	Δ SRMR
**M1**	Configural Invariance	98.37	82	0.98	0.97	0.04	[0.001, 0.060]	0.04	9781.13	9836.81	–	–	–		
**M2**	Metric Invariance	120.13	103	0.98	0.97	0.03	[0.001, 0.060]	0.06	9760.88	9804.63	M2 vs. M1	21.76*	0.00	−0.01	0.02
**M3**	Scalar Invariance	141.80	112	0.97	0.96	0.04	[0.014, 0.061]	0.06	9764.56	9803.19	M3 vs. M1	43.43*	−0.01	0.00	0.02
											M3 vs. M2	21.67*	−0.01	0.01	0.00

*Non-significant differences (p < 0.01).

In contrast, measurement invariance could not be established for the ESEM model (Model 8; cf. Table 7). Non-significant differences in χ^2^ was apparent between the metric- and configural- (Δχ^2^ = 18.59), as well as between the scalar- and configural models (Δχ^2^ = 41.10) (*p* > 0.01). However, a statistically significant difference in χ^2^ was found between the scalar and metric models (Δχ^2^ = 21.56; *p* < 0.01). Further, changes in CFI between the scalar and configural models were higher than the suggested cut-off (ΔCFI < 0.01). Therefore, measurement invariance could not fully be established for the ESEM model. Partial invariance was not considered given that it provides biased interpretations of latent mean comparisons (De Beuckelaer and Swinnen, 2018). The ESEM model was therefore not considered for further comparisons.

**TABLE 7:** Invariance testing between the different genders for the ESEM Model (Model 8).

	Model	χ ^2^	*df*	CFI	TLI	RMSEA		SRMR	AIC	aBIC	Model Comparison	Δχ ^2^	Δ CFI	Δ RMSEA	Δ SRMR
**M1**	Configural Invariance	108.34	86	0.97	0.96	0.04	[0.001, 0.063]	0.04	9783.09	9836.50	–	–	–		
**M2**	Metric Invariance	126.93	106	0.98	0.97	0.04	[0.001, 0.057]	0.05	9761.69	9803.73	M2 vs. M1	18.59*	0.01	0.00	0.01
**M3**	Scalar Invariance	153.44	116	0.96	0.95	0.05	[0.023, 0.064]	0.06	9768.20	9804.56	M3 vs. M1	45.10*	−0.01	0.01	0.02
											M3 vs. M2	26.51	−0.02	0.01	0.01

*Non-significant differences (p < 0.01).

### Latent Mean Comparisons

Given that the B-ICM-CFA-1 model showed invariance, further investigation into the differences between males and females are permitted. As such, latent mean comparisons were estimated. With males as the reference group, the results showed that females scored statistically significantly higher on the unstandardized fitted mean on interest (Δ*M* = 0.40; *SE* = 0.18; *p* < 0.05) and perseverance (Δ*M* = 0.75; *SE* = 0.16; *p* < 0.5). However, no significant differences in overall grit (G-Factor) could be established (Δ*M* = −0.20; *SE* = 0.18; *p* > 0.05).

### Concurrent Validity^4^

In order to establish concurrent validity, the best fitting model (B-ICM-CFA-1) which showed to invariance, was used to determine the relationship between the General Grit Factor, the two specific factors (perseverance of effort and consistency of interest) and Task Performance. We used a structural model to establish concurrent validity through estimating a regressive path between perseverance of effort and consistency of interest and task performance. The model fitted the data significantly (χ^2^ = 256.67; CFI = 0.93; TLI = 0.92; RMSEA = 0.05 [CI: 0.043−0.063]; SRMR = 0.05). The regression paths showed that the general grit factor (*B* = 0.60; *SE*: 0.10; *p* < 0.05), and the two specific factors, perseverance of effort (*B* = 0.30; *SE*: 0.12) and consistency of interest (*B* = 0.17; *SE*: 0.08) were significant predictors of task performance. These factors declared 45.1% of the total variance in task performance (*R*^2^ = 0.45; *p* < 0.05). The results therefore show support that the bifactor model of the Grit-O is concurrently valid.

## Discussion

The purpose of this study was to investigate the psychometric properties of Duckworth et al. (2007) original Grit-O scale using both traditional ICM-CFA and ESEM models within a European context (Netherlands). Specifically, the aim was to determine the instrument’s factorial validity (ICM-CFA: first, second and bi-factor vs. ESEM: first order ESEM, and Bifactor ESEM), measurement invariance across genders, and internal consistency. The results showed that both a traditional Bifactor ICM-CFA structure (consisting of a general factor of grit and two specific factors relating to consistency of interest and perseverance of effort), as well as a less restrictive two factor ESEM model could be used to validly and reliably measure grit within this context. However, only the Bifactor ICM-CFA model showed to be invariant between genders and that females score higher on perseverance and interest. However, no statistically significant differences between genders could be found for general grit. In contrast, invariance could not be established for the ESEM model, which implies that it may produce biased estimates when trying to compare genders. Finally, the results showed that the Bi-Factor model was a significant predictor of Task Performance within the current sample.

### Factorial Validity of the Grit-O Scale

Motivated by the increased usage of the Grit-O scale in the mass media and popular psychology self-development books, coupled with the lack of strong psychometric evidence supporting its use, the first aim of this study was to investigate its factorial validity and to determine whether an ICM-CFA or ESEM factorial solution may be preferred. With the exclusion of the bifactor ICM-CFA model with two specific factors (B-ICM-CFA-1: Model 6), the results showed that none of the originally reported ICM-CFA factor structures of the Grit-O scale should be considered to be self-evident. Neither a first- nor hierarchical second order one- (Duckworth et al., 2007), two- (Duckworth and Quinn, 2009; Christensen and Knezek, 2014; Tyumeneva et al., 2017), or three factorial model solution (Kim and Lee, 2015) could sufficiently be confirmed within the current sample. Further, despite showing excellent data-model fit, ICM-CFA bifactor model with interest, perseverance and industriousness as specific factors (B-ICM-CFA-2: Model 7) failed to produce a significant general grit factor. This implies that ICM-CFA factorial models, assuming a strict differentiation between the components of grit, may not be appropriate within the current context, or critiques as to the construct validity of grit (in general) might be valid (Credé, 2018).

Although there is a clear conceptual and theoretical distinction between different permutations of the sub-factors of grit, our ICM-CFA factorial models show that grit may not be appropriately computed (or viewed) as a mere sequential aggregation of lower-level facet scores. Credé et al. (2017) argued that for grit to be seen as a higher order construct, two conditions need to be met: (a) facets need to be strongly and uniformly correlated, and (b) a higher order model shows better/worse fit than a lower order model. Neither the Models 2 and 3 nor Models 4 and 5 completely satisfied either of these criteria. Between the first order and second order factorial models, no distinction could be made in model fit. Secondly, the inter-factorial correlations between the factors within the various permutations were lower than 0.5 and thus not uniformly or strongly correlated. Therefore, the construct validity and the hierarchical nature of grit by these traditional ICM-CFA models are questionable. As such, future studies should carefully consider all factor structure permutations when employing the Grit-O scale.

Similarly, despite providing excellent fit, two out of the three ESEM factors were not appropriate for the data. The Bifactor ESEM (B-ESEM: Model 10) model with perseverance and interest as two specific factors failed to produce a significant general grit factor. Further, the three factor ESEM model (Model 9) produced significant cross-loadings on the majority of the items on the industriousness and perseverance subscales. Although mathematically permitted, this model was rejected from further analyses.

However, both the two-specific factor ICM-CFA Bifactor model and the two factor ESEM model showed excellent data-model fit and produced appropriate factor loadings. The Bifactor model showed that both the general and specific components, perseverance and interest, may have unique explanatory power and provide relevant and unique information. The factor inter-correlations also show that perseverance and interest are uniformly correlated in the presence of the General Grit Factor. Taken together this means that within the bifactor structure, grit should not be seen as a function of the interplay between perseverance and interest but should rather be viewed as an omni-present factor that is separate from perseverance and interest. The mean scores of both the general and the specific factors could therefore be used as valid indicators within this model. This is in contrast to the findings of the bi-factor estimation of Disabato et al. (2018), who indicated that only the overall score of grit should be considered. Our findings support the ide that both the general grit factor as wall as the two specific factors add independent value that is unrelated to their factorial interactions.

The results further showed that the ESEM solution provided the best possible data-model fit, while taking into consideration factor-loadings. This suggests that a less restrictive model may be more beneficial when considering the estimation of grit. Small cross-loadings between factors may result in better differentiation between factors and result in better model fit (Wang and Wang, 2012). Further, the ESEM model may prove to be more appropriate than the ICM-CFA approaches and may produce a more accurate or realistic representation of perseverance and interest within real-world data (Morin et al., 2013). Given that ESEM has never been applied to the Grit-O scale, it is difficult to compare results to other studies.

### Internal Consistency of the Grit-O Scale: ESEM vs. B-ICM-CFA

Determining the best fitting factorial solution of the Grit-O scale allowed for further investigation into the internal consistency of the instrument. At least two studies concentrated on the lower-bound level of internal consistency of the Grit-O scale and reported significant variations (between 0.65 and 0.89) across samples and between contexts (Duckworth et al., 2007; Kim and Lee, 2015). The current study reported acceptable levels of internal consistency at both the lower-bound (Cronbach’s alpha > 0.70) and upper-bound limits (ρ > 0.70) (Wang and Wang, 2012) for the two specific factors and the general factor within the Bifactorial solution of the Grit-O. Further, the point-estimate composite reliability (ρ_*esem*_ > 0.70) measure of Raykov and Shrout (2002) showed that the two facets within the ESEM solution also showed to be reliable. These results suggested that both the Bifactor and ESEM solution of the Grit-O was reliable within the current context.

### Measurement Invariance Between Genders

Confirming the factor structure and internal consistency of the Grit-O scale allowed for further investigation into the configural (i.e., factorial equivalence), metric (i.e., similarity in item loadings and factor structures), and scalar (i.e., determining similar intercepts) invariances between males and females for both the Bifactor and ESEM factorial solutions. The study showed that the Bifactor model is invariant between genders, while not in the ESEM model. Within the ESEM solution, significant differences between the invariance models were found, which indicates that when the ESEM solution is employed, it may provide biased comparisons between genders.

In contrast, within the Bifactor solution, the results indicated full configural, metric and scalar invariances between the two genders. First, the configural results showed that males and females conceptualized both the general (general grit) and two factors of the Grit-O scale (perseverance and interest) in a similar fashion. Second, the metric invariance results indicated a non-significant difference between males and females, suggesting that the two gender groups interpreted the items of the Grit-O in the same way (He and Van de Vijver, 2012). Finally, the scalar invariance results indicated that both males and females perceived the Grit-O in the same way, meaning that grit is measured in a similar way between genders (He and Van de Vijver, 2012). In other words, individuals (whether male or female) who had the same score on the latent variables obtained the same score on the measured items (Van de Schoot et al., 2012). Thus, in the context of the current sample, the bifactor solution of the Grit-O scale can be used to methodically and systematically compare, contrast and discriminate between the grit of males and females.

Latent mean comparisons between genders on the ICM-CFA Bifactor model showed that females reported higher levels of interest and perseverance than males. However, no significant differences in overall grit could be established. This implies that females may be more inclined to show more long-term interest in their goals, and therefore may be more determined to pursue such, even in the face of adversity. This result supports the findings of Christensen and Knezek (2014), who reported that females tend to be grittier than males. This is in contrast to Duckworth and Quinn (2009), who argued that grit is a universal trait that does not differ between genders.

### Concurrent Validity: Grit vs. Task Performance

To determine the concurrent validity of the Grit-O Scale, the study established the relationship between the general grit factor and the specific components thereof (perseverance of effort and consistency of interest) and task performance. The results indicated that in this sample, all three the components of the bifactor model were significantly related to task performance. However, our results indicated that the overall grit factor was more strongly associated with task performance than the two specific factors. This implies that overall grit plays a more important role in motivating individuals to perform, than each individual factor (Vogelsang, 2018). The dynamic interaction between an individual’s long-term interests and their ability to push through difficult scenarios, may lead individuals to perform better because they see how tasks relate to long term goals (Disabato et al., 2018).

### Limitations and Recommendations

The present study is not without its limitations. First, the Grit-O scale is a transparent self-report measure, which is a limitation in terms of socially desirable answers. Participants might have responded positively to some of the items in a way that might make them look good.

Second, the validation study employed a cross-sectional research design, which in itself is limited. As grit seems to remain relatively stable across time and situation (Von Culin et al., 2014), it might be fruitful to conduct longitudinal studies to explore how stable grit actually is over time and whether its mean level will remain consistent over time. Grittier individuals may not perform any better than the less grittier ones when first starting a job, but their performance may increase over time as a function of their ambition to succeed.

Third, the study employed a convenience sampling strategy to obtain respondents in a specific, albeit unique, region in the Netherlands. Within the current study, there is a large proportion of German speaking nationals present, which is not representative of the entire Netherlands. It is, however, roughly in line with the demographics of the working population within the Twente region. Further, the age ranges and level of education of participants may also not be aligned to the overall population. However, this skewed distribution limits the interpretative frame and scope of the current study. We urge readers to be cautions when interpreting the findings. In effect, this approach severely limits the generalizability of the study beyond that of the sampled population.

Fourth, it is important to note that these ICM-CFA factorial permutations may be possible in other studies. Within the current sample only one of the incremental fit cut-off criteria was slightly violated (i.e., TLI < 0.90) (Wang and Wang, 2012) and therefore the model was rejected. Brown (2006) argued that TLI is sensitive to both model complexity and sample size. As such, larger samples and a more complex model may yield slightly better TLI values in future studies. As the current study strictly adhered to the CFA guidelines and cut-off criteria proposed by Wang and Wang (2012), no modifications to the models were made to inflate model complexity. Unlike Kim and Lee (2015) in the English version and Schmidt et al. (2017) in the German version, who modified the factor/item structure in order to improve the data fit of the Grit-O scale, the current study did not allow for (a) items and error terms to be correlated, (b) items to be parcelled, or (c) slopes/intercepts to be constrained. This significantly reduced the complexity of the model, which in effect could have decreased model fit (Kline, 2005; Brown, 2006). Although not recommended in clinical trials, Bentler and Chou (1987) argued that incorporating correlated error terms or parceling items may be considered when “real world data” is employed and the sample size is small.

Finally, although the study assessed measurement invariance between genders, the sample distribution was skewed toward females (69.1%). Even though the sample was large enough to compute the configural, scalar, and metric invariances between the genders, a larger sample of males might have influenced the results. Future studies should aim to obtain an equal distribution of males and females for invariance testing.

## Conclusion

Despite thorough attempts to replicate and compute every possible theoretical-factorial structure of the Grit-O, it would seem as though only a Bi-Factor structure, with one general and two specific factors, emerged as the best fitting model. The bifactorial solution seems to be the best fitting, most reliable and the only model that could discriminate between genders within the current sample, despite other models showing superior model fit. Researchers, practitioners and the general populous aiming to employ the Grit-O scale within the Netherlands as a means to assess grit, should be wary of its straight forward use.

## Data Availability Statement

The raw data supporting the conclusions of this article is available upon request to any qualified researcher.

## Ethics Statement

The authors declare that they strictly adhered to the APA guidelines on ethical research practices. The University of Twente’s ethics committee reviewed and approved the project.

## Author Contributions

LZ conceptualized the study, conducted the statistical analyses and drafted the methods section, analyses section and results section of the manuscript. CO aided in the conceptualisation of the manuscript, and drafted the literature review. LR aided in attending to the revisions of the manuscript.

## Conflict of Interest

The authors declare that the research was conducted in the absence of any commercial or financial relationships that could be construed as a potential conflict of interest.

## Footnotes

1 As of 31 May 2019, Duckworth’s (2016) book is still listed as the number 4 bestselling business book by the *New York Times.*

2 Hereafter referred to as perseverance.

3 Hereafter referred to as interest.

4 Concurrent validity for the ESEM model was not actively reported in text, because it was eliminated during the invariance phase. However, the results are reported in Appendix A for interested readers.

## Appendix A

### Concurrent Validity of the Esem Model

Concurrent validity of the ESEM model was estimated through SEM. A structural model to employed, estimating a regressive path between perseverance of effort and consistency of interest and task performance. The model fitted the data significantly (χ^2^ = 254.96; CFI = 0.94; TLI = 0.92; RMSEA = 0.05 [CI: 0.042−0.062]; SRMR = 0.05). The regression paths showed that only perseverance of effort (*B* = 0.64; *SE*: 0.05) was a significant predictor of task performance declaring 42.3% of the total variance there in (*R*^2^ = 0.423; *p* < 0.05). Consistency of interest, on the other hand, did not (*B* = 0.04; *SE*: 0.06).
