# Corrigendum: The Psychometric Properties of the Grit-O Scale Within the Twente Region in Netherlands: An ICM-CFA vs. ESEM Approach

**DOI:** 10.3389/fpsyg.2020.623096

**Published:** 2020-11-20

**Authors:** Llewellyn E. van Zyl, Chantal Olckers, Lara C. Roll

**Affiliations:** ^1^Department of Industrial Engineering, University of Eindhoven, Eindhoven, Netherlands; ^2^Optentia Research Focus Area, North-West University (VTC), Vanderbijlpark, South Africa; ^3^Department of Human Resource Management, University of Twente, Enschede, Netherlands; ^4^Institut für Psychologie, Goethe University, Frankfurt am Main, Germany; ^5^Department of Human Resource Management, University of Pretoria, Pretoria, South Africa; ^6^Department of Applied Psychology, Lingnan University, Tuen Mun, Hong Kong

**Keywords:** confirmatory factor analysis, exploratory structural equation modeling, grit, invariance testing, psychometric properties, validity

In the published article, there was an error in affiliation **5**. Instead of “**5**”, it should be “**6**”.

In the published article, there was an error in affiliation **6**. Instead of “**6**”, it should be “**5**”.

The authors apologize for this error and state that this does not change the scientific conclusions of the article in any way. The original article has been updated.

