# Corrigendum: The Psychometric Properties of the Grit-O Scale Within the Twente Region in Netherlands: An ICM-CFA vs. ESEM Approach

**DOI:** 10.3389/fpsyg.2021.688081

**Published:** 2021-05-06

**Authors:** Llewellyn E. van Zyl, Chantal Olckers, Lara C. Roll

**Affiliations:** ^1^Department of Industrial Engineering, University of Eindhoven, Eindhoven, Netherlands; ^2^Optentia Research Focus Area, North-West University (VTC), Vanderbijlpark, South Africa; ^3^Department of Human Resource Management, University of Twente, Enschede, Netherlands; ^4^Institut für Psychologie, Goethe University, Frankfurt am Main, Germany; ^5^Department of Human Resource Management, University of Pretoria, Pretoria, South Africa; ^6^Department of Applied Psychology, Lingnan University, Tuen Mun, Hong Kong

**Keywords:** confirmatory factor analysis, exploratory structural equation modeling, grit, invariance testing, psychometric properties, validity

In the original article, there was an omission. In the original published manuscript, a description of the Task Performance Subscale used to establish concurrent validity was omitted from the Measures Section.

A correction has been made to the section *Materials and Methods, Measures*. The following paragraph is added after the description of the Grit-O Scale, before the section *Statistical Analyses*:

The *Task Performance Subscale* of the Individual Work Performance Scale developed by Koopmans et al. (2013) was employed to measure task performance by means of seven items on a 6-point Likert scale ranging from 1 (“Never”) to 6 (“Always”). An example of an item is: “I kept in mind the results that I had to achieve in my work.” Van Zyl et al. (2019) found acceptable levels of internal consistency for the instrument with a Cronbach's alpha level of 0.88.

The authors apologize for this error and state that this does not change the scientific conclusions of the article in any way. The original article has been updated.
